# Men and Horses

**Published:** 1873-02

**Authors:** 


### MEN AND HORSES.

In 1788, a horse of the finest Arabian blood, named Messenger, was imported into New York; his form was superb, his muscular power extraordinary, with a vitality, an endurance, and a spirit which made him the wonder of his time. By a system of breeding with the best, his qualities have been intensified in his progeny up to the present time. Millions of dollars have been expended in improving the breed of horses, and the success has been steady and uniformly encouraging. The general profit consists in this, that it costs no more to keep an inferior horse than a superior one, while the latter has twice the endurance, and can do double the work, consequently is worth more. In 1858, Flora Temple was sold for $8,000. Damsee brought $11,000. Four years later, $25,000 was paid for Pocahontas. Five years ago, Mr. Bonner gave for Dexter, $33,000; and within a week the cable brings the news from England that the stallion "Blair Athol," brought at auction over $65,000. Dexter has trotted a mile in two minutes and seventeen and a quarter seconds. It is but a few years ago that sportsmen were wondering if the mile would ever be passed over in the "teens," for then the best horses never came down to 2–20, that is, two minutes and twenty seconds. Now, if beauty of form, and endurance and spirit can be improved upon by scientific management as to horses, it should not be questioned that men could arrive at greater health and more vigor of body and brain by proper attention in bringing together those who have vigorous health, strong minds, and high morals.

